# 70-fs mode-locked erbium-doped fiber laser with topological insulator

**DOI:** 10.1038/srep19997

**Published:** 2016-01-27

**Authors:** Wenjun Liu, Lihui Pang, Hainian Han, Wenlong Tian, Hao Chen, Ming Lei, Peiguang Yan, Zhiyi Wei

**Affiliations:** 1 Beijing National Laboratory for Condensed Matter Physics, Institute of Physics, Chinese Academy of Sciences, Beijing 100190, China; 2 State Key Laboratory of Information Photonics and Optical Communications, School of Science, P. O. Box 91, Beijing University of Posts and Telecommunications, Beijing 100876, China; 3 Shenzhen Key Laboratory of Laser Engineering, College of Optoelectronic Engineering, Shenzhen University, ShenZhen, 518060, China

## Abstract

Femtosecond optical pulses have applications in optical communication, astronomical frequency combs, and laser spectroscopy. Here, a hybrid mode-locked erbium-doped fiber (EDF) laser with topological insulator (TI) is proposed, for the first time to our best knowledge. The pulsed laser deposition (PLD) method is employed to fabricate the fiber-taper TI saturable absorber (TISA). By virtue of the fiber-taper TISA, the hybrid EDF laser is passively mode-locked using the nonlinear polarization evolution (NPE), and emits 70 fs pulses at 1542 nm, whose 3 dB spectral width is 63 nm with a repetition rate and transfer efficiency of 95.4 MHz and 14.12%, respectively. Our experiments indicate that the proposed hybrid mode-locked EDF lasers have better performance to achieve shorter pulses with higher power and lower mode-locking threshold in the future.

Mode-locked fiber lasers are powerful sources of ultra-short pulses^1^,^2^,^3^. They have the advantages over solid-state pulse lasers in the system robustness, beam quality, pumping efficiency, power scalability and easy operation, and have some applications in such fields as spectroscopy, ultrafast science and telecommunications^4^,^5^,^6^,^7^,^8^. Among them, EDF lasers have attracted much attention, and passively mode-locked techniques, such as the SA and NPE, have been suggested to achieve the mode-locking operation in EDF lasers^9^,^10^,^11^,^12^,^13^,^14^.

The NPE technique, which has such advantages as ultra-short pulse width, high stability, simple structure and compact size, is a powerful one to generate the ultra-short pulses^15^,^16^. In 2010, a dechirped pulse width of 37.4 fs at a 225 MHz repetition rate has been reported with 10% transfer efficiency in the EDF laser^12^. In 2015, the shortest 34.3 fs pulse generation dechirped from a NPE mode locked EDF laser has been demonstrated with 5.5% transfer efficiency^13^. However, in the NPE mode locked EDF lasers, the transfer efficiency is usually low, and most of them have the high mode-locking threshold^17^,^18^.

On the other hand, some SAs, such as semiconductor saturable absorber mirrors (SESAMs), carbon nanotubes, graphene, and TI, have been used to investigate the mode-locked fiber lasers^19^,^20^,^21^,^22^,^23^,^24^,^25^,^26^,^27^,^28^,^29^. In those SAs, TIs have exhibited the ultra large saturable intensity and modulation depth, and have been the object of extensive experimental research in recent years^30^,^31^,^32^,^33^,^34^,^35^. Sub-170-fs pulses with 34-nm full width at half-maximum (FWHM) have been obtained in an all-fiber EDF laser mode-locked by Sb_2_Te_3_ SA, and the maximum output power is 5.34 mW with a repetition rate of 26 MHz^36^. And then, another EDF laser based on an evanescent field interaction with the Sb2Te3 has been presented, the shortest pulses of 125 fs have been obtained with the FWHM, repetition rate, average output power of 37 nm, 22.4 MHz and 1 mW, respectively^37^. While for those compact all-fiber lasers, the disadvantage of them is the low repetition rate, which is typical limited to 20–50 MHz. In addition, the average output power of them is low. Thus, the compact fiber lasers with high repetition rate, high output power, high transfer efficiency, low mode-locking threshold and narrow pulse duration are needed.

In this investigation, a new design of the EDF laser is demonstrated. The mode-locked scheme of the EDF laser is a hybrid scheme devised by incorporating the NPE and fiber-taper TI: Sb_2_Te_3_ SA technique. The NPE adopted here is intended to shape ultra-short pulses in the dispersion management (DM) soliton regime. To avoid excessive intra-cavity nonlinear side effects of the EDF laser, the fiber-taper TI: Sb_2_Te_3_ SA is used to perform nonlinear filtering of the pulse amplitude. Attributing to the tapered fiber, the Sb_2_Te_3_ is not exposed to high optical power, so that it allows the fiber-taper TI: Sb_2_Te_3_ SA in the EDF laser to work at the high power regime. At the same time, the fiber-taper TI: Sb_2_Te_3_ SA facilitates pulse initiation, and can suppress the parasite waves separated from the main pulses. The hybrid mode-locked scheme theoretically permits generating shorter pulses with the advantages of higher average power and lower mode-locking threshold compared to the traditional mode-locked mechanism. With this means, 70 fs optical pulses with a repetition rate of 95.4 MHz and 63 mW output power have been generated, which is to our best knowledge the shortest pulses and highest output power ever produced by any fiber lasers with TISA.

## Results

### Fabrication of fiber-taper TI: Sb_2_Te_3_ SA

**Figure 1:**
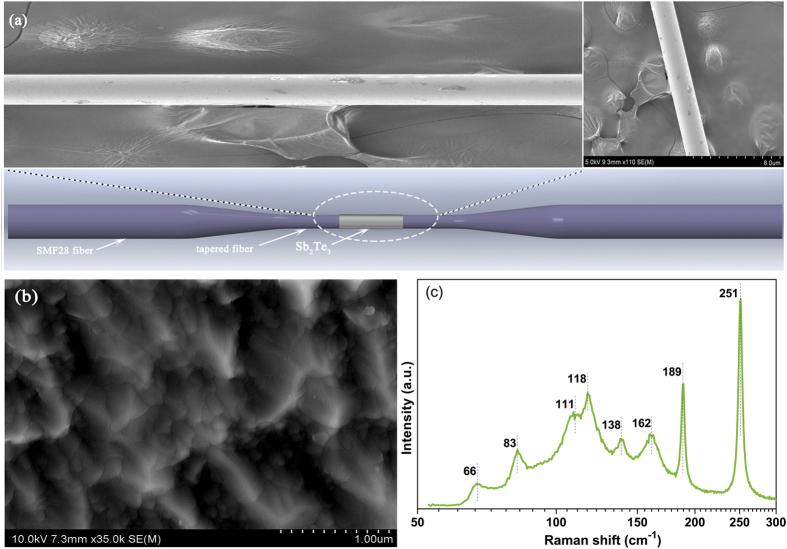
(**a**) Schematic illustration of the fiber-taper TI: Sb_2_Te_3_ SA and the corresponding scanning electron microscope image of the taper waist; (**b**) Film surface of the fiber-taper TI: Sb_2_Te_3_ SA; (**c**) Raman spectrum of Sb2Te3 on the fiber-taper TI: Sb_2_Te_3_ SA.

The SA is fabricated by depositing the TI: Sb_2_Te_3_ on the tapered fiber. The tapered fiber is fabricated by fiber-tapering equipment with the SMF-28 fiber. The length of the fiber-taper is about 2 mm, and the waist is about 18 μm. During the fabrication of fiber-taper TI: Sb_2_Te_3_ SA, a Nd: YAG laser (λ = 1550 nm) is employed in the pulsed laser deposition (PLD) system as an ablation source with 10 Hz repetition rate. The single pulse energy is 200 mJ, and the average power of the laser is 2 W. The target is Sb_2_Te_3_, and the Vacuum pressure of the chamber is fixed at 10^−4^ pa during the deposition procedure. The deposition time is 60 minutes. Figure 1(a,b) show the scanning electron microscope (SEM) diagram of the fiber taper. Layers of Sb_2_Te_3_ are deposited on a tapered fiber, which enables the interaction between the evanescent fields of propagating beam. That developed technology allows for controlling the length of a deposited material, and thereby for changing the length of light-TI interaction^31^. Raman spectra is measured by using a Raman spectrometer (LabRAM HR Evolution) with a laser at 514 nm. A typical laser power of 5.6 mW is used to excite the Raman scatting. Figure 1(c) shows the measured Raman spectrum. The lines observed at 66, 111, and 162 cm^−1^ are well related to A_1g_ and E_g_ modes of Sb_2_Te_3_. The lines at 189 and 251 cm^−1^ are related to Sb_2_O_3_. All those peaks were known to exist in the Sb_2_Te_3_ crystal^36^.

**Figure 2:**
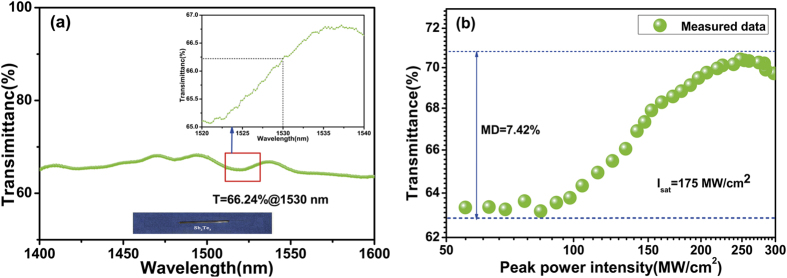
(**a**) Measured linear absorption of the fiber-taper TI: Sb_2_Te_3_ SA at the level of 65%±2%, and the real device graph; (**b**) The corresponding nonlinear saturable absorption with a modulation depth of 7.42%, and the saturable intensity of 175 MW/cm^2^.

The insertion loss (IL) of the fiber-taper TI: Sb_2_Te_3_ SA is measured to be ~2 dB. An amplified spontaneous emission (ASE) source (Glight, 1250 nm~1650 nm) and optical spectrum analyzer are used to measure the device’s linear absorption from 1400 nm to 1600 nm. As shown in Fig. 2(a), the linear transmission is characterized by a very flat profile at the level of 65% ± 2%, which indicates that Sb_2_Te_3_ is a promising optical material. A femtosecond pulse is used to measure the nonlinear saturable absorption with the pulse duration of 650 fs, central wavelength of 1562 nm, and repetition rate of 22.5 MHz. The corresponding result is shown in Fig. 2(b), which gives a saturable intensity is at of 175 mW/cm^2^, and a modulation depth Δ*α* of 7.42%. The interaction between the evanescent field leaks to the fiber clad with the Sb_2_Te_3_ surface. The fiber-taper TI: Sb_2_Te_3_ SA is spliced to the DM laser resonator based on the EDF fiber.

### Experimental setup

**Figure 3:**
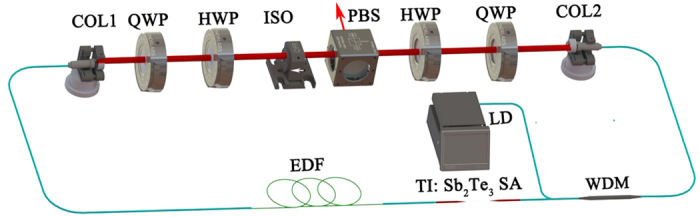
Configuration of the hybrid mode-locked EDF laser. COL, collimator; QWP, quarter wave plate; HWP, half wave plate; ISO, polarization-dependent isolator; PBS, polarization beam splitter; WDM, wavelength-division multiplexer; TI: Sb_2_Te_3_ SA, topological insulator Sb_2_Te_3_ saturable absorber.

The schematic diagram of the hybrid mode-locked EDF laser is illustrated in Fig. 3. The ring cavity consisted of a highly gain EDF (Liekki 110 −4/125) with absorption coefficient of 250 dB/m at 980 nm, two HI1060 fiber pigtail of 980 nm/1550 nm wavelength division multiplexer (WDM), two SMF-28 leading fiber collimators and one polarization beam splitting (PBS). Two half wave plates (HWP) and quarter wave plates (QWP) are engaged to achieve different polarization sates, and the polarization-dependent isolator (ISO) is used to force the unidirectional operation in the oscillator cavity. The ISO plays the double role of an isolator and a polarizer such that light leaving the isolator is linearly polarized. TI: Sb_2_Te_3_ SA is a saturable absorber device with fiber-taper. The EDF is pumped by a 976 nm laser diode (LD) with maximum output power of 500 mW. At last, the HWP and QWP can optimize the polarization state to launch into the gain EDF.

The total length of the ring cavity is about 2.11 m. The fiber section consists of a 340-mm-long, highly doped EDF, a 465-mm-long fiber for the WDM, and the leading fiber for the two collimators (SMF28, 250 and 385 mm long). The output pulses from the intra-cavity PBS are measured by an optical spectrum analyzer (Yokogawa AQ6315A), 1-GHz photo-detector and a 250-MHz oscilloscope (Tektronix TDS 714L), RF spectrum analyzer (Agilent E4407B), and an optical intensity autocorrelator ( Femtochrome, FR-103XL).

### Experimental results

**Figure 4:**
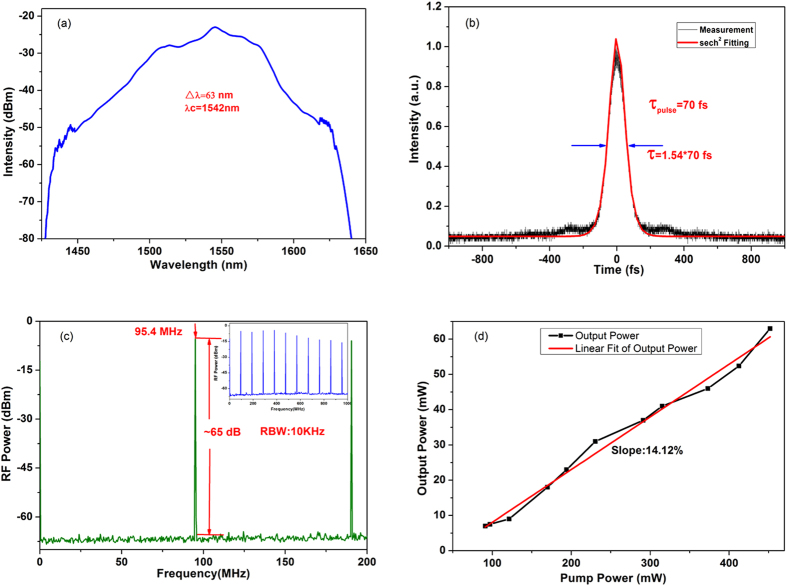
Experimental results of the hybrid mode-locked EDF laser. (**a**) Optical spectrum of the generated pulses, (**b**) Intensity autocorrelation trace, (**c**) Radio frequency (RF) spectrum of the mode-locked laser, (**d**) Laser output power as a function of the pump power.

In the experiment, the EDF laser starts to operate in mode-locked regime when the pump power is increased to 91 mW. This mode-locking threshold is relatively low, which is mainly due to the fiber-taper TI: Sb_2_Te_3_ SA. As shown in Fig. 4(a), the optical spectrum of mode-locked pulses is centered at 1542 nm, and the 3 dB spectral width is 63 nm. Figure 4(b) shows the measured intensity autocorrelation trace of the output pulses together with secant hyperbolic-fitting curve. The pulse duration is 70 fs. The radio frequency (RF) spectrum of the laser is depicted in Fig. 4(c). The fundamental cavity repetition rate is 95.4 MHz. The electrical signal to noise ratio (SNR) is better than 65 dB measured with 10 kHz resolution bandwidth (RBW), demonstrating the mode-locking state is quite stable. The maximum power of the direct output pulse at an available pump power of 447 mW is about 63 mW, which exhibits a high transfer efficiency of 14.12% as shown in Fig. 4(d). When the pump power is increased to 447 mW, the mode-locking operation become unstable, and the fluctuation of spectrum is obvious. However, the stable mode-locking operation is observed again when the pump power decreases from 447 mW. Thus, the mode-locking remains stable for pump powers in the range from 91 mW to 447 mW.

**Figure 5:**
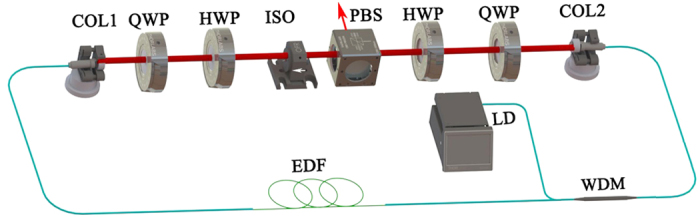
Configuration of the mode-locked EDF laser without the fiber-taper TI: Sb_2_Te_3_ SA.

In order to analyze the effect of the fiber-taper TI: Sb_2_Te_3_ SA, we remove this device, and measure the performance of the mode-locked EDF laser without the fiber-taper TI: Sb_2_Te_3_ SA, which is illustrated in Fig. 5. With 36 mW output power of the 447 mW pump power, the 3-dB spectral width and pulse duration are, respectively, measured as 55.7 nm and 83 fs with a frequency of 128 MHz. The electrical SNR is about 76 dB. The transfer efficiency of the fiber lasers is about 10% as shown in Fig. 5(d). When the pump power is 315 mW, the output power is 31.5 mW. When the pump power is lower than 315 mW, the mode-locking operation is unstable. However, the stable mode-locking operation is observed again when the pump power increases to 315 mW.

**Figure 6:**
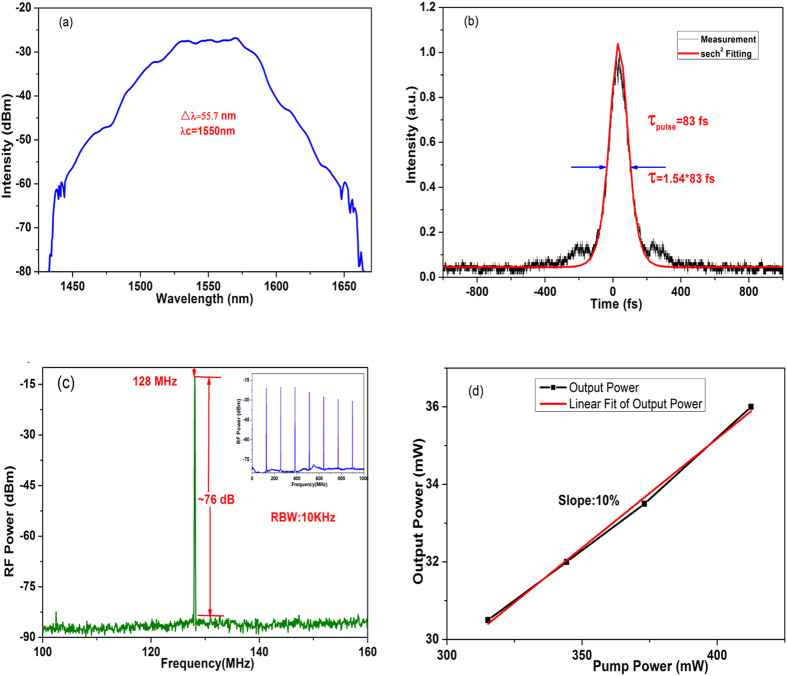
Experimental results of the mode-locked EDF laser without the fiber-taper TI: Sb_2_Te_3_ SA. (**a**) Optical spectrum of the generated pulses, (**b**) Intensity autocorrelation trace, (**c**) Radio frequency (RF) spectrum of the mode-locked laser, (**d**) Laser output power as a function of the pump power.

From the above experimental results in Figs 3 and 5, we summarize the corresponding parameters as shown in Table 1. We can find that the output power of the hybrid mode-locked EDF laser is higher than the one without the fiber-taper TI: Sb_2_Te_3_ SA. When the intra-cavity power increases as the stability of the mode-locking, the evanescent field around tapered fiber is easily observed with green light while optical pulses confined into other parts of fiber core could not be seen. This phenomenon indicates that the fiber-taper TI: Sb_2_Te_3_ SA shows strong nonlinearity with the high power, and is not destroyed by the thermal accumulation. Compared to Figs 4(a) and 6(a), the 3-dB spectral width of the hybrid mode-locked EDF laser gets wider, and the pulse duration is shorter (see Figs 4(b) and 6(b)). Because the waist of the tapered fiber becomes thinner, the nonlinear effect is enhanced. Besides, in the real part of nonlinear refractive index of the TI thin films, they show stronger nonlinear effects. Moreover, the fiber-taper TI: Sb_2_Te_3_ SA enables the interaction between the evanescent fields of propagating beam, which impact on the TI thin films. Thus, the 3-dB spectral width gets wider.

When optical pulses pass through the fiber-taper TI: Sb_2_Te_3_ SA, the loss of the edge is greater than the central part. Thus, optical pulses are narrow during the process of passing through the fiber-taper TI: Sb_2_Te_3_ SA. Moreover, the hybrid mode produces an average power of 63 mW at the output port while the output power is 36 mW in Fig. 5. Those results confirm that the fiber-taper TI: Sb_2_Te_3_ SA performs its intended functions with the nonlinear attenuation of the pulse amplitude. The intra-cavity power is reduced by the fiber-taper TI: Sb_2_Te_3_ SA and gradually built up in the EDF without excessive nonlinear side effects. Then the amplified pulse is compressed in the SMF-28 fiber to a short pulse. Because of the presence of the fiber-taper TI: Sb_2_Te_3_ SA, the length of the cavity increases, and the repetition frequency reduces as shown in Figs 4(c) and 6(c). Besides, a mode-locking threshold is 91 mW in Fig. 3, while the mode-locking threshold is 315 mW in Fig. 5, which indicates that the mode-locking threshold of the hybrid mode-locked EDF laser is lower. This is due to the presence of the fiber-taper TI: Sb_2_Te_3_ SA, which is helpful for the mode-locked fiber lasers to enhance the self-starting capability. Moreover, the transfer efficiency in Fig. 4(d) is higher than that in Fig. 6(d). In the imaginary part of nonlinear refractive index of the TI thin films, it is acted as the saturable absorption, which can reshape and narrow the pulses in the cavity of EDF lasers. On the other hand, the power density of the cavity is higher due to the existence of the fiber-taper TI: Sb_2_Te_3_ SA, and the nonlinear effects also increase. Therefore, in our hybrid mode-locked EDF laser, the mode-locking threshold is low, the pulse duration is short, and the repetition rate is high.

In order to evaluate the long term stability of mode locking, the EDF laser has been working 7 days continuously, and the spectrum remains reasonably stable. Additionally, if we do not change the position of the polarization controller knobs after the stable mode-locked operation, the constructed laser undergoes self-starting each time when the pump is retuned into the power range. Self-starting behavior of the system is observed even if the laser undergoes transportation between laboratories or is unpowered for 7 days.

## Discussion

We presented the first demonstration of the hybrid mode-locked EDF laser based on the NPE and fiber-taper TI: Sb_2_Te_3_ SA. The pulse duration is 70 fs, which is the shortest pulse duration generated from any fiber lasers with TISA. Besides, the optical spectrum of mode-locked pulses has been centered at 1542 nm, and the 3 dB spectral width has been 63 nm with a frequency of 95.4 MHz. The maximum power of the direct output pulses at the pump power of 447 mW has been about 63 mW, which exhibits a high transfer efficiency of 14.12%. Moreover, the mode-locking threshold of our laser is low, and the electrical SNR has been better than 65 dB. The obtained results show that the fiber-taper TI: Sb_2_Te_3_ SA can act as an effective components for ultra-short pulse generation in fiber lasers. Furthermore, the proposed hybrid mode-locked EDF laser can reduce the mode-locked threshold, and enhance the transfer efficiency in generating shorter pulses with higher power, which is easier to be mode-locked and more robust than the EDF laser mode-locked NPE. By optimizing the optical performance of the components of hybrid mode-locked EDF lasers, we anticipate that one can break the record of NPE mode-locked EDF lasers to generate sub-30-fs pulses with high average output power in the future work, thus further expanding the application space of the mode-locked EDF laser.

## Additional Information

**How to cite this article**: Liu, W. *et al.* 70-fs mode-locked erbium-doped fiber laser with topological insulator. *Sci. Rep.* **6**, 19997; doi: 10.1038/srep19997 (2016).

## Figures and Tables

**Table 1:** Summary of the parameters with hybrid mode-locked EDF lasers.

Mode-locking scheme	Spectral Width (nm)	Pulse Duration (fs)	Output Power (mW)	Transfer Efficiency	Mode-locking Threshold (mW)
Hybrid (NPE + fiber-taper TI: Sb_2_Te_3_ SA)	63	70	63	14.12%	91
NPE	55.7	83	36	10%	315
